# Oxidative phosphorylation promotes vascular calcification in chronic kidney disease

**DOI:** 10.1038/s41419-022-04679-y

**Published:** 2022-03-11

**Authors:** Jia Shi, Yi Yang, Ya-Nan Wang, Qing Li, Xue Xing, An-Ying Cheng, Xiao-Na Zhan, Jie Li, Gang Xu, Fan He

**Affiliations:** Department of Nephrology, Tongji Hospital, Tongji Medical College, Huazhong University of Science and Technology, 1095 Wuhan, Jiefang Ave China

**Keywords:** Calcification, End-stage renal disease

## Abstract

Metabolism has been reported to associate with the progression of vascular diseases. However, how vascular calcification in chronic kidney disease (CKD) is regulated by metabolic status remains poorly understood. Using a model of 5/6 nephrectomy, we demonstrated that the aortic tissues of CKD mice had a preference for using oxidative phosphorylation (OXPHOS). Both high phosphate and human uremic serum-stimulated vascular smooth muscle cells (VSMCs) had enhanced mitochondrial respiration capacity, while the glycolysis level was not significantly different. Besides, 2-deoxy-d-glucose (2-DG) exacerbated vascular calcification by upregulating OXPHOS. The activity of cytochrome *c* oxidase (COX) was higher in the aortic tissue of CKD mice than those of sham-operated mice. Moreover, the expression levels of COX15 were higher in CKD patients with aortic arch calcification (AAC) than those without AAC, and the AAC scores were correlated with the expression level of COX15. Suppressing COX sufficiently attenuated vascular calcification. Our findings verify the relationship between OXPHOS and calcification, and may provide potential therapeutic approaches for vascular calcification in CKD.

## Introduction

Chronic kidney disease (CKD) is a rising health problem worldwide, and the prevalence of CKD was 9.2% in the world [1]. CKD patients are often diagnosed with vascular calcification. As the estimated glomerular filtration rate (eGFR) declines, the prevalence of vascular calcification increases [2]. A study revealed that 78.3% of hemodialysis patients have vascular calcification [3]. Vascular calcification is related to a higher cumulative incidence of myocardial infarction and heart failure, in parallel with the risk of cardiovascular and all-cause mortality [4]. Vascular calcification happens in both the intima and media, and the predominant vascular pathology in the CKD population is medial calcification [5, 6]. Although patients with CKD also develop intima calcification, studies revealed that mortality is linked more to medial calcification [6]. Therefore, the determination of the molecular mechanisms of medial calcification is important.

Vascular smooth muscle cells (VSMCs), the dominating components of the artery’s media, are known to be the major cells regulating the progress of medial calcification [5, 7]. In calcified blood vessels, the contractile VSMCs convert to an osteo-/chondrogenic phenotype, with the upregulation of Runx2, Bmp2, Sox9, and Alpl expression [8, 9]. High blood phosphate is a crucial risk factor for cardiovascular mortality in CKD [10]. Besides, high phosphate is a strong inducer of medial calcification, which is linked to osteogenic differentiation of VSMCs [11]. However, the mechanisms of how osteogenic differentiation is regulated in VSMCs during CKD remained ill-defined.

Glucose has been implicated as the main energy source for VSMCs [5]. Recently, studies have reported that glucose metabolism participates in the progression of vascular calcification. Pyruvate dehydrogenase kinase (PDK) is an enzyme which mediates the glucose metabolic shift from oxidative phosphorylation (OXPHOS) to glycolysis [5]. Inhibition of PDK4 by dichloroacetate abrogated vitamin D3-induced calcification in rodent models [12]. However, in the same study, 2-deoxy-d-glucose (2-DG), which is considered a better option than other glycolysis inhibitors as it inhibits the first crucial step in glycolysis [13], significantly reduced the production of lactate while exacerbated calcium deposition in the VSMCs [12]. To our knowledge, previous study did not investigate the role of glucose metabolism under CKD conditions. As discrepancies may be caused by differences in cell types and assay conditions, it is necessary to investigate the glucose requirement for medial calcification in CKD models.

In addition to glycolysis, mitochondria fuel metabolic processes through their generation of ATP. Mitochondrial respiratory complexes are essential to OXPHOS, where electron transfer to oxygen is combined with ATP production [14]. A previous study has found that suppressing the activity of mitochondrial respiratory complex I and II could ameliorate neointima hyperplasia after arterial injury [15]. Whether OXPHOS participates in the progression of vascular calcification remains unclear. The mechanism of vascular calcification in CKD and its relationship with OXPHOS warrant investigation.

In this study, we investigated the glucose requirement for VSMCs calcification under the uremic condition both in vivo and in vitro. We tested the role of OXPHOS in vascular calcification, and explored that whether interfering with OXPHOS helps alleviate calcification.

## Results

### Glucose metabolism was enhanced in calcified VSMCs and aortas

**Fig. 1:**
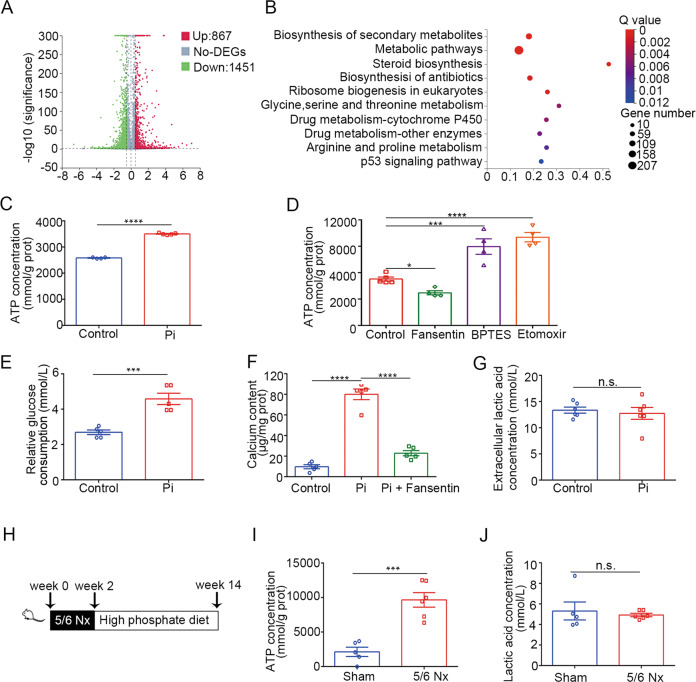
Glucose metabolism was enhanced in calcified VSMCs and aortas. **A**, **B** Transcriptome analysis of control or high phosphate-treated MOVAS were performed. **A** Volcano plot of gene expression changes. **B** The top ten enriched KEGG pathways in the transcripts. **C** ATP concentration of HASMC (unpaired *t* test; control *n* = 4, high phosphate *n* = 5). **D** ATP concentration of HASMC treated with high phosphate (one-way ANOVA; *n* = 4). **E** Relative glucose consumption of HASMC (unpaired *t* test; *n* = 5). **F** Calcium contents in HASMC (one-way ANOVA; *n* = 5). **G** Extracellular lactic acid concentration of HASMC (unpaired *t* test; *n* = 6). **H** Scheme. Mice underwent a two-step, 5/6 nephrectomy, followed by high phosphate diet for 3 months. **I** ATP concentration of aortas of mice (unpaired *t* test; sham *n* = 5, 5/6 nephrectomy *n* = 6). **J** Lactic acid concentration of abdominal aortas of mice (unpaired *t* test; sham *n* = 5, 5/6 nephrectomy *n* = 6). ***P < 0.001, ****P < 0.0001, n.s. not significant.

As shown in Supplementary Fig. S1A, B, Alizarin red staining and calcium content detected a notable calcification of VSMCs after treated with high phosphate. Besides, the expression of osteogenic transformation marker was upregulated (Supplementary Fig. S1C). To identify transcriptional signature of high phosphate-treated VSMCs, we performed transcriptomic analysis. Differential expression analysis was conducted comparing high phosphate to the control group, yielding a group of 867 significantly upregulated genes and 1451 downregulated genes (Fig. 1A). The KEGG pathway assessment of DEGs revealed that metabolic pathways were enriched in high phosphate-induced VSMCs (Fig. 1B). Next, we analyzed the ATP concentration of high phosphate-treated VSMCs, which was significantly higher than the control group (Fig. 1C). To access the relative contribution of major nutrients to intracellular ATP homeostasis, we used fasentin, etomoxir and BPTES to inhibit glucose uptake, fatty acid, and glutamine metabolism, respectively. The treatment of fasentin inhibited the production of ATP in high phosphate-treated VSMCs, whereas BPTES or etomoxir induced ATP generation, which implied that inhibition of fatty acid or glutamine metabolism promotes mitochondrial ATP production under high phosphate conditions (Fig. 1D). More importantly, the rate of glucose consumption was significantly greater in VSMCs treated with high phosphate, and suppression of glucose uptake by fasentin attenuated calcification, indicating that the glucose metabolism is enhanced upon high phosphate stimulation (Fig. 1E, F). We also examined the concentration of lactate, the product of glycolysis, which had no difference between two groups (Fig. 1G).

In order to induce CKD, 5/6 nephrectomy was performed in mice and high phosphate diet was fed (Fig. 1H). The levels of serum creatinine, blood urea nitrogen, and serum phosphate increased significantly in CKD mice compared with sham-operated mice fed with a normal phosphate diet (Supplementary Fig. S2A). More apparent calcification of the aortas, as identified by Alizarin red staining and calcium content, was found in CKD mice (Supplementary Fig. S2B, C). In addition, the increased expression of osteogenic transformation markers (Runx2, Sox9, and ALPL) were upregulated in the aortas of CKD mice (Supplementary Fig. S2D). Meanwhile, the ATP concentration was higher in the aortas of CKD group compared to sham-operated mice (Fig. 1I), while no obvious difference in lactate production could be found between the two groups (Fig. 1J). These results indicated that glucose metabolism is enhanced with the development of vascular calcification.

### OXPHOS was increased in high phosphate-induced calcification

**Fig. 2:**
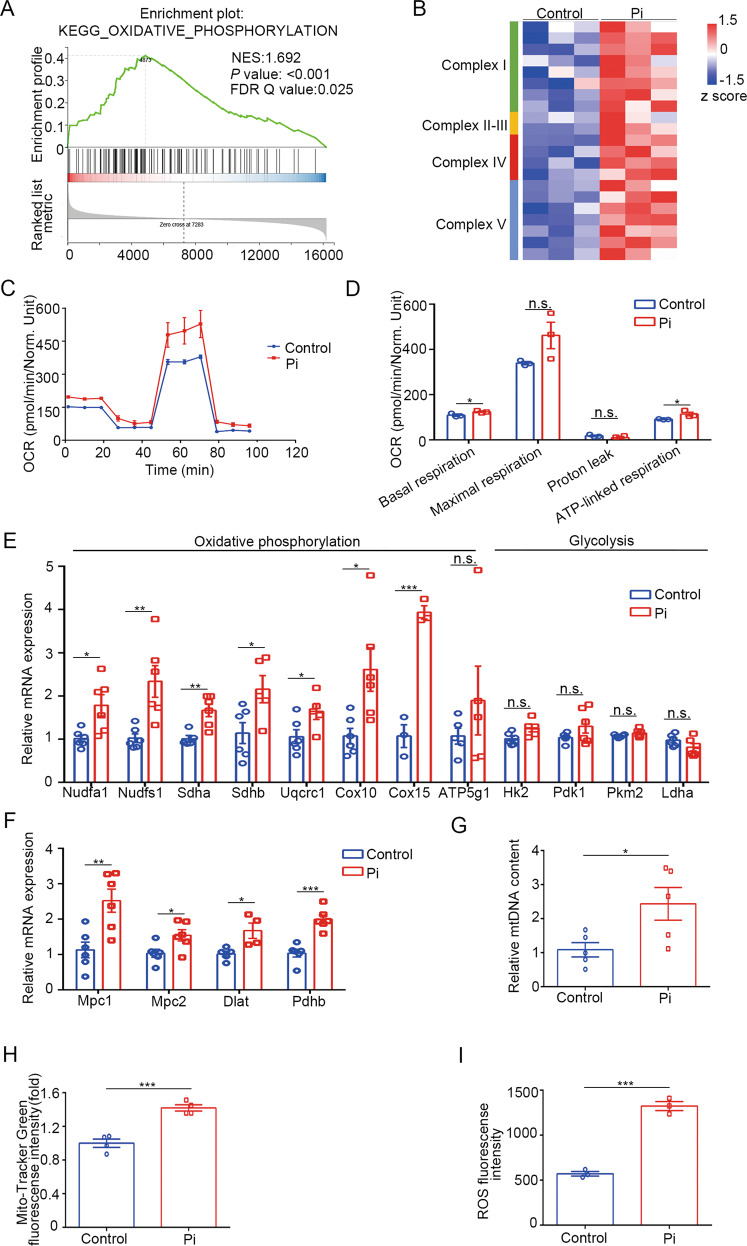
OXPHOS was increased in high phosphate-induced calcification. **A** GSEA plots of KEGG pathways showed that OXPHOS was enriched in the high phosphate group. **B** Heatmap of selected enriched terms from GSEA plots. **C** OCRs were measured in control or high phosphate-treated HASMC (*n* = 3). **D** Basal respiration, maximum respiration, proton leak, and ATP-linked respiration in OCRs were analyzed (unpaired *t* test; *n* = 3). **E** Relative mRNA expression of OXPHOS-related and glycolysis-related genes in HASMC as detected by RT-qPCR (unpaired *t* test; *n* = 5–6). **F** Relative mRNA expression of genes related to the influx of pyruvate into mitochondria (unpaired *t* test; *n* = 5–6). **G** Relative mtDNA content (unpaired *t* test; *n* = 5). **H** Measurement of the mitochondrial mass (unpaired *t* test; *n* = 4). **I** Measurement of the ROS level (unpaired *t* test; *n* = 3). *P < 0.05, **P < 0.01, ***P < 0.001, n.s. not significant.

To explore the key signaling pathways involved in metabolic pathways, GSEA enrichment was performed. The results showed that OXPHOS was significantly enriched (Fig. 2A). Cluster analysis of OXPHOS-related genes showed that they were increased in VSMC treated with high phosphate (Fig. 2B). To determine the OXPHOS changes, we examined Oxygen consumption rate (OCR), which enables direct quantification of mitochondrial respiration. The OCR, including basal respiration and ATP-linked respiration, was higher in high phosphate-stimulated VSMCs (Fig. 2C, D). Besides, high phosphate-treated VSMCs exhibited higher expression of OXPHOS-related genes, while glycolysis-related genes remained at a similar level during differentiation (Fig. 2E). We then wondered about the underlying mechanisms of enhanced OXPHOS rather than glycolysis during VSMCs calcification. The expression level of genes facilitating the influx of pyruvate into mitochondria for OXPHOS were increased in high phosphate-treated VSMCs (Fig. 2F), indicating that increased OXPHOS may be mediated by increased pyruvate influx into mitochondria.

Mitochondrial biogenesis is the basis for cellular energy metabolism under stress, and reactive oxygen species (ROS) are the byproduct of OXPHOS metabolism in cells [16]. Thus, we compared the mtDNA copy number, mitochondrial contents, and ROS levels in VSMCs with or without high phosphate treatment. As shown in Fig. 2G, H, the relative mtDNA copy number and Mito-Tracker green fluorescence intensity were significantly higher in high-phosphate-treated VSMCs. In addition, the ROS level was also higher in high-phosphate-treated VSMCs (Fig. 2I).

### Enhanced OXPHOS in VSMCs during uremic conditions

**Fig. 3:**
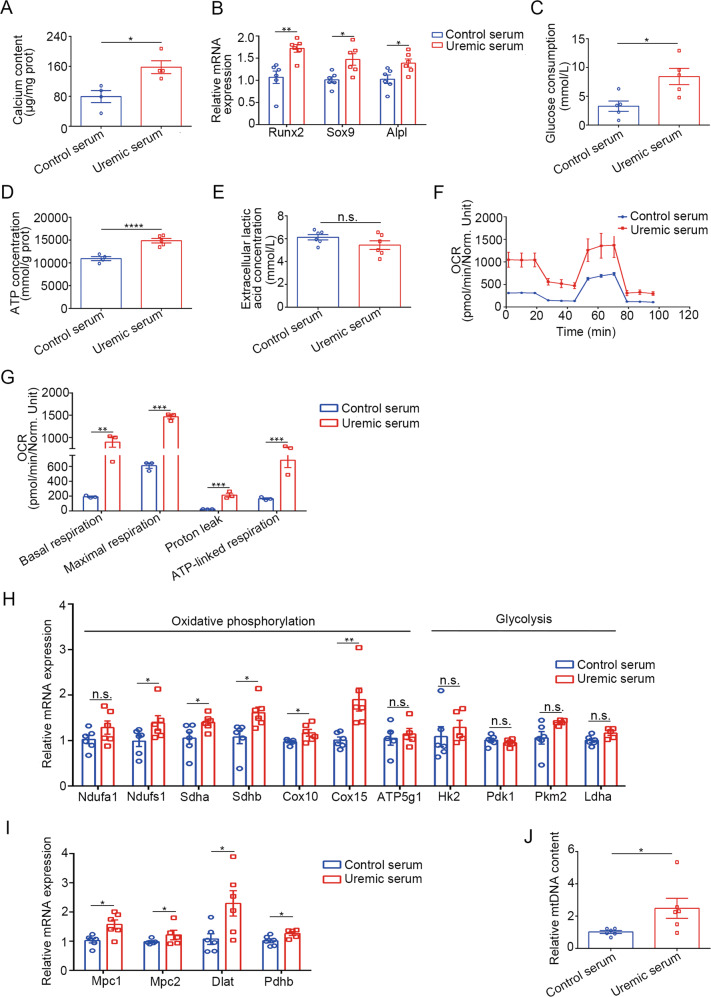
OXPHOS was enhanced in uremia-related calcification. HASMC were cultured in medium treated with serum from healthy control or uremic patients. **A** Total calcium content in HASMC (unpaired *t* test; *n* = 4). **B** Relative mRNA expression of Runx2, Sox9, ALPL in HASMC as detected by RT-qPCR (unpaired *t* test; *n* = 6). **C** Relative glucose consumption of HASMCs (unpaired *t* test; *n* = 5). **D** ATP concentration of HASMC (unpaired *t* test; *n* = 5). **E** Extracellular lactic acid concentration of HASMC (unpaired *t* test; *n* = 6). **F** OCRs were measured in HASMC (*n* = 3). **G** Basal respiration, maximum respiration, proton leak, and ATP-linked respiration in OCRs were analyzed (unpaired *t* test; *n* = 3). **H** Relative mRNA expression of genes related to the influx of pyruvate into mitochondria (unpaired *t* test; *n* = 5–6). **I** Relative mRNA expression of OXPHOS-related and glycolysis-related genes in HASMCs as detected by RT-qPCR (unpaired *t* test; *n* = 6). **J** Relative mtDNA content (unpaired *t* test; *n* = 6). *P < 0.05, **P < 0.01, ***P < 0.001, ****P < 0.0001, n.s. not significant.

To identify the effect of uremic serum, VSMCs were treated with serum from healthy participants (control serum) and uremic patients (uremic serum). As shown in Fig. 3A, the calcium content in human aortic smooth muscle cells (HASMCs) treated with uremic serum was higher than those treated with control serum, and the real-time quantitative polymerase chain reaction (RT-qPCR) results confirmed the osteogenic differentiation (Fig. 3B). Consistent with the results in high phosphate-treated VSMCs, the glucose consumption and ATP concentration were significantly higher in VSMCs with uremic serum treatment, and no difference in the lactate production was found between the two groups (Fig. 3C–E). Metabolic flux analysis in uremic serum-stimulated VSMCs showed increased mitochondrial respiration, characterized by an obvious increase in basal respiration, maximal respiration, proton leak, and ATP-linked respiration (Fig. 3F, G). Moreover, HASMCs treated with uremic serum showed higher level of OXPHOS-related genes and the genes related to the influx of pyruvate into mitochondria (Fig. 3H, I). The mtDNA content was also higher in HASMCs treated with uremic serum (Fig. 3J).

### 2-DG accelerated high phosphate-induced calcification by upregulating OXPHOS

**Fig. 4:**
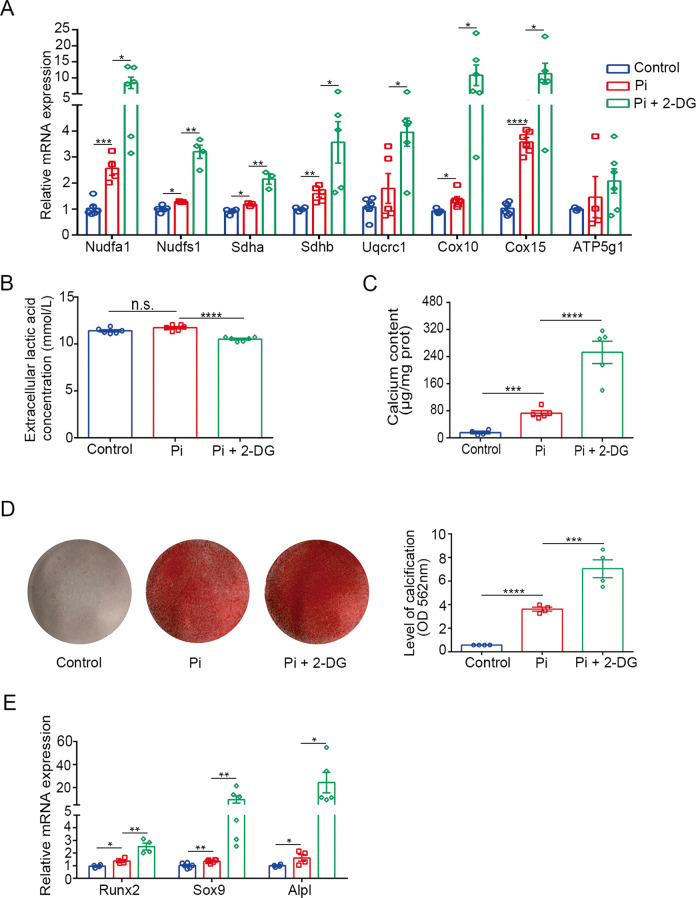
2-DG accelerated high phosphate-induced calcification by upregulating OXPHOS. **A** Relative mRNA expression of OXPHOS-related genes in HASMC as detected by RT-qPCR (one-way ANOVA; *n* = 4–6). **B** Extracellular lactic acid concentration of HASMC (one-way ANOVA; *n* = 6). **C** Total calcium content in HASMC (one-way ANOVA; *n* = 5). **D** Representative images and quantification of Alizarin red staining of HASMC (one-way ANOVA; *n* = 4). **E** Relative mRNA expression of Runx2, Sox9, ALPL in HASMC as detected by RT-qPCR (one-way ANOVA; *n* = 4–6). *P < 0.05, **P < 0.01, ***P < 0.001, ****P < 0.0001.

2-DG is an inhibitor of the predominantly glycolytic protein, hexokinase 2 [17]. A previous study has shown that 2-DG could increase OXPHOS level [16], we presumed that 2-DG may inhibit glycolysis and increase mitochondrial OXPHOS. To provide support for this hypothesis, we measured the production of lactic acid and the expression of OXPHOS-related genes in VSMCs. The RT-qPCR results revealed that the expression of mitochondrial respiratory complex I–IV apparently increased after treatment with 2-DG (100 μM) (Fig. 4A). Besides, 2-DG significantly decreased lactate production in high phosphate-treated VSMCs (Fig. 4B). After 2-DG treatment, both calcium contents and Alizarin red staining results revealed that the calcification significantly exacerbated (Fig. 4C, D). Consistently, the osteogenic transformation of high phosphate-induced VSMCs was enhanced (Fig. 4E).

### Cytochrome *c* oxidase (COX) is involved in enhanced OXPHOS of calcified aortas and VSMCs

**Fig. 5:**
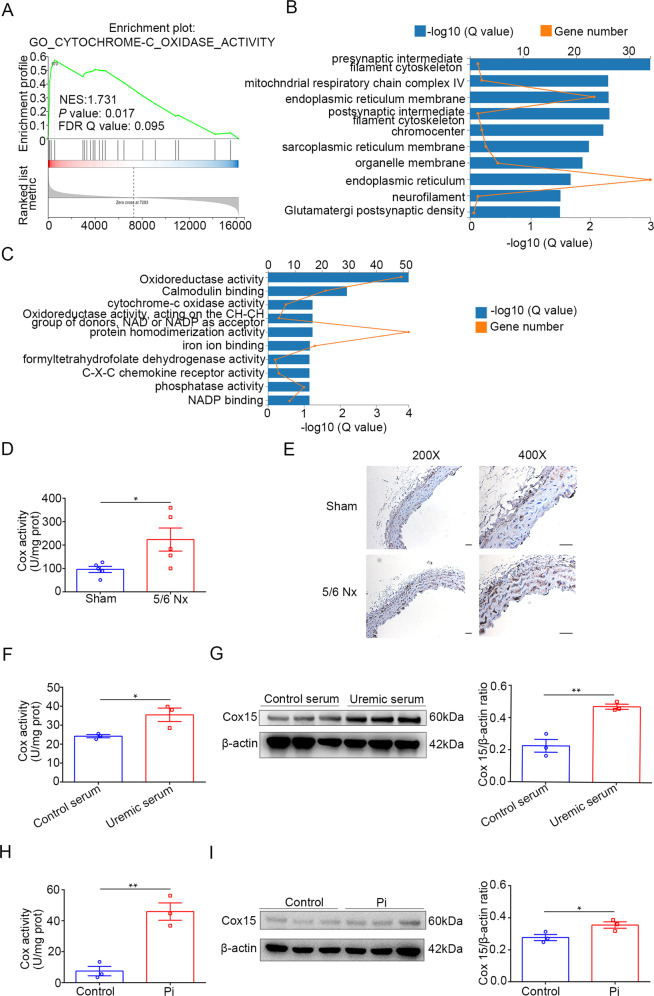
Cytochrome *c* oxidase is involved in enhanced OXPHOS of calcified aortas and VSMCs. **A** GSEA plots of GO pathways showed that COX was enriched in the high phosphate group. **B** The top ten enriched organelles of GO cellular component terms in the transcripts. **C** The top ten enriched GO molecular function terms in the transcripts. **D** COX activities measured in mice (unpaired *t* test; *n* = 5). **E** Immunohistochemistry of aortic COX15 expression in sham and 5/6 nephrectomy mice. **F** COX activities measured in HASMC treated with serum from healthy control or uremic patients (unpaired *t* test; *n* = 3). **G** Western blot and its quantification analysis in HASMC treated with serum from healthy control or uremic patients (unpaired *t* test; *n* = 3). **H** COX activities measured in HASMC treated with control or high phosphate (unpaired *t* test; *n* = 3). **I** Western blot and its quantification analysis in HASMC treated with control or high phosphate (unpaired *t* test; *n* = 3). *P < 0.05, **P < 0.01. Scale bar = 20 µm.

To further investigate the molecular mechanisms that control the metabolic status of the calcifying VSMCs, we investigated several clusters that were upregulated in calcified VSMCs. GSEA enrichment revealed that COX, also known as mitochondrial complex IV, which is the final and rate-limiting step of the OXPHOS [18], was significantly enriched (Fig. 5A). Consistently, COX was notably upregulated in calcified VSMCs in GO molecular function and cellular component analysis (Fig. 5B, C). We also found that COX15 expressed at the highest degree in both high phosphate-induced and uremic serum-stimulated VSMCs (Figs. 2E and 3H). In CKD mice, the COX activity is significantly higher than sham group (Fig. 5D). Besides, the immunohistochemistry results revealed that more COX15 expressed predominantly in the media of the aortas in the CKD mice (Fig. 5E). Similarly, the COX activity and protein level of COX15 were upregulated in VSMCs treated with uremic serum compared to those treated with control serum (Fig. 5F, G). Furthermore, the COX activity and protein level of COX15 of high phosphate-treated VSMCs were remarkably increased (Fig. 5H, I). These hints prompted us to consider whether COX is one of the major regulators that enhance OXPHOS utilization in calcified VSMCs.

### Inhibition of COX attenuated high phosphate-induced calcification

**Fig. 6:**
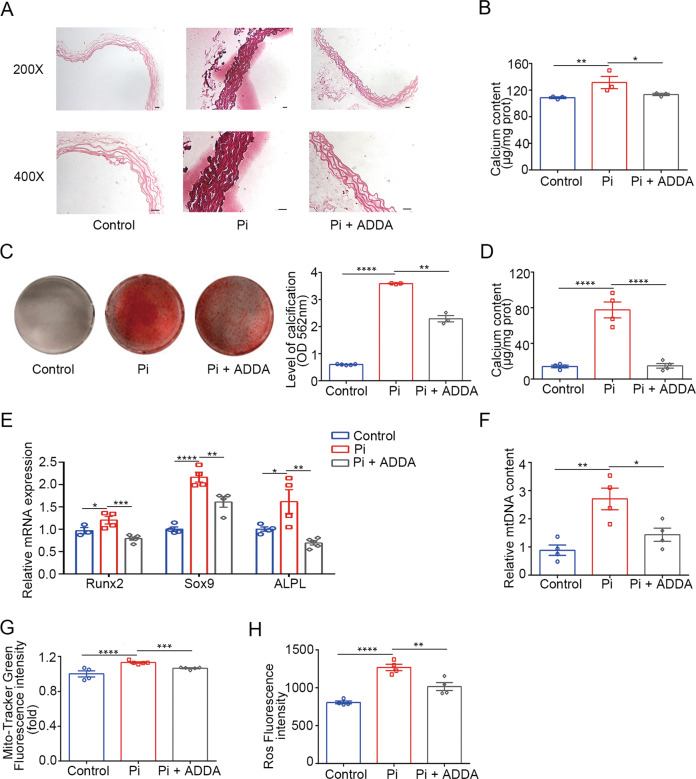
Inhibition of cytochrome *c* oxidase attenuated high phosphate-induced calcification. **A**, **B** Aortic rings were cultured in control or high phosphate medium with or without ADDA 5 hydrochloride supplementation. **A** Representative images of Alizarin red staining of aortic rings from mice. **B** Total calcium content in aortic rings (one-way ANOVA; *n* = 3). **C** Representative images and quantification of Alizarin red staining of HASMCs (one-way ANOVA; *n* = 3–5). **D** Total calcium content in HASMC (one-way ANOVA; *n* = 4). **E** Relative mRNA expression of Runx2, Sox9, ALPL in HASMC as detected by RT-qPCR (one-way ANOVA; *n* = 4). **F** Relative mtDNA content (unpaired *t* test; *n* = 4). **G** Measurement of the mitochondrial mass (one-way ANOVA; *n* = 4–5). **H** Measurement of the ROS level (one-way ANOVA; *n* = 4). *P < 0.05, **P < 0.01, ***P < 0.001, ****P < 0.0001. Scale bar = 20 µm.

To assess the COX function in calcification, we assessed whether pharmacological inhibition of COX could attenuate the calcification of vascular tissue ex vivo. Therefore, aortic rings were isolated from the thoracic aortas of mice and incubated with high phosphate in the presence or absence of ADDA 5 hydrochloride, a non-competitive specific inhibitor of COX [19]. Application of ADDA 5 hydrochloride (25 nM) significantly decreased calcification of the aortic rings (Fig. 6A, B). Consistently, ADDA 5 hydrochloride treatment attenuated high phosphate-induced calcification of VSMCs (Fig. 6C, D). Molecular analyses by RT-qPCR confirmed the significant suppression of osteogenic differentiation markers (Fig. 6E). In addition, the increased mtDNA content, Mito-Tracker Green intensity, and ROS levels in high phosphate-treated VSMCs were significantly attenuated by ADDA 5 hydrochloride treatment (Fig. 6F–H).

### Increased expression of COX15 in artery tissues of CKD patients

**Fig. 7:**
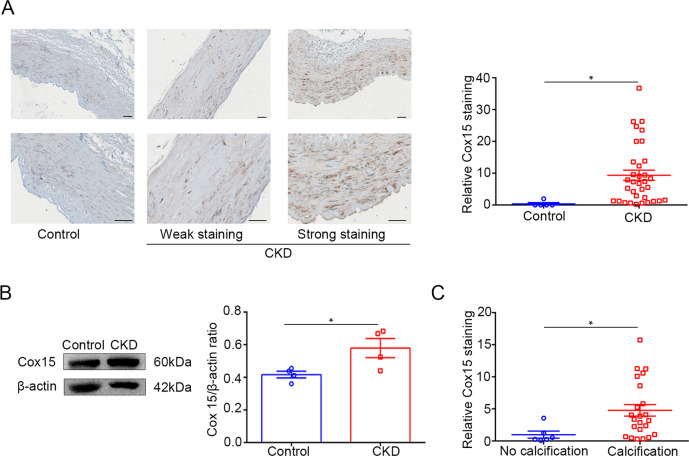
The expression of Cox15 is enhanced in the artery of patients with CKD. **A** Representative images and quantification of immunohistochemistry staining of COX15 in arteries of patients with or without CKD (unpaired *t* test; Control *n* = 5, CKD *n* = 34). **B** Western blot and its quantification analysis in arteries of patients with or without CKD (unpaired *t* test; *n* = 4). **C** Comparison of the intensity of COX15 expression in CKD patients with aortic arch calcification and those without aortic arch calcification (unpaired *t* test; no calcification *n* = 6, calcification *n* = 23). *P < 0.05. Scale bar = 50 µm.

To investigate the role of COX15 in CKD patients, we conducted an immunohistochemical study in cases from our center. The intensity of COX15 expression was notably higher in the CKD group compared to the control group (Fig. 7A). Consistently, results from western blot confirmed that COX15 was upregulated in artery tissues in the CKD group (Fig. 7B).

In our cohort, all cases were positive for COX15 protein in different degrees, 20 of 34 (58.8%) cases showed strong positive staining for COX15 protein, and 14 cases showed weak positive staining. According to the intensity of the expression of COX15, we further divided CKD patients into two groups. The relationship between COX15 staining levels and clinicopathological factors are summarized in Table 1. No significant difference in the characteristics, including blood urea nitrogen, creatinine, and parathyroid hormones, was observed between the two groups. Among 34 patients in our cohort, chest computed tomography was conducted in 29 patients (85.3%), which was used to measure aortic arch calcification (AAC). It is worth noting that the AAC scores were significantly higher in CKD patients with strong COX15 staining (*P* = 0.035). Moreover, we compared the intensity of COX15 staining between CKD patients with and without AAC. As shown in Fig. 7C, the expression levels of COX15 staining of patients with AAC were higher compared with those without calcification.

**Table 1:** Comparison of baseline demographics and clinical characteristics of CKD patients with weak and strong COX15 expression.

Parameters	Weak staining	Strong staining	*P*
*N*	14	20	–
Age, years	51.0 ± 3.5	56.7 ± 2.3	0.161
Male sex, *n* (%)	6 (42.9)	15 (75.0)	0.080
Hypertension, *n* (%)	10 (71.4)	19 (95.0)	0.056
Laboratory findings			
Hemoglobin, g/L	91.0 ± 7.2	95.61 ± 4.5	0.572
Platelet, 10^9^/L	222.0 ± 29.2	179.8 ± 15.9	0.184
Blood urea nitrogen, mmol/L	21.4 ± 3.2	26.0 ± 1.6	0.165
Serum creatinine, μmol/L	850.4 ± 121.3	922.0 ± 79.9	0.610
Lactic acid, mmol/L	387.9 ± 45.2	459.8 ± 29.6	0.173
Adjusted calcium, mmol/L	2.3 ± 0.1	2.3 ± 0.1	0.382
Potassium, mmol/L	2.0 ± 0.2	2.0 ± 0.1	0.939
1,25(OH) D-vitamin, ng/L	14.0 ± 2.0	15.7 ± 1.8	0.538
Parathyroid hormone, ng/L	256.7 (179.8–305.9)	236.5 (153.4–500.7)	0.656
Triglycerides, mmol/L	2.2 ± 0.4	2.3 ± 0.4	0.864
HDL-cholesterol, mmol/L	1.0 ± 0.1	0.8 ± 0.1	0.103
LDL-cholesterol, mmol/L	2.1 ± 0.2	2.1 ± 0.2	0.794
AAC, *n* (%)^†^	7 (58.3)	16 (94.1)	**0.019**
AAC score^†^	1.1 ± 0.3	1.9 ± 0.2	**0.035**

*AAC* aortic arch calcification, *HDL* high-density cholesterol, *LDL* low-density cholesterol.

Values are expressed as mean ± standard error of the mean, median (25th–75th percentile), or *n* (%). Significant data are in bold. ^†^5 data missing.

## Discussion

In this study, we uncovered a previously unrecognized role of OXPHOS in vascular calcification in CKD and acquired some interesting findings. First, this is the first study to directly measure the contribution of OXPHOS to VSMCs treated with serum from uremic patients, which showed that OXPHOS plays a pivotal role in the progression of calcification in CKD. Second, the data presented herein document that COX, a key component of the mitochondrial respiratory chain, expressed higher in arteries from CKD patients and mice than those from the control group. Moreover, the artery of CKD patients with AAC expressed higher level of COX15, and the AAC scores were correlated with the level of COX15 staining. Finally, we found that inhibition of COX15 attenuated high phosphate-induced calcium deposition both ex vivo and in vitro, which provided potential therapeutic strategies for vascular calcification. Together, our data underline the importance of OXPHOS for vascular calcification.

Increasing evidence has shown that abnormal metabolic signaling participates in the progression of calcification. In this study, we applied three inhibitors affecting the major pathways of energy production, and found that glucose is the main energy substrate for VSMCs. Glucose metabolism makes key functions in vascular reactivity [20]. As the major components of artery’s medial layer, VSMCs exhibit unusually high rates of glucose utilization [21]. Our results indicated that calcified VSMCs exhibit more robust glucose metabolism and suppression of glucose uptake attenuated calcification. Current research on the role of glycolysis remains uncertain. A recent study observed an abnormal increase in glycolytic flux in periostin-induced calcification [22]. Besides, Ma et al. reported that the expression of the glycolysis enzyme was enhanced in vitamin D-induced calcification [12]. However, in our study, the lactate production showed no significant difference both in aortas of CKD mice compared to the sham group, and the expression of glycolysis-related genes in uremic serum-treated VSMCs was comparable to the control group. The reason for this discrepancy may be explained by the different calcification model used. Interestingly, Ma et al. observed that inhibition of glycolysis by 2-DG exacerbated calcification, which is consistent with the results in our study [12]. They suggested that glycolysis may serve as a metabolic adaption to ensure the survival of differentiated VSMCs [12]. Future studies are required to test the specific role of glycolysis in vascular calcification in the CKD model.

It has been known that the major source of intracellular ATP is mitochondrial OXPHOS. Mitochondria are crucial in the regulation of cellular metabolism and growth [15], it has been reported that OXPHOS provides an appropriate basis for tumor progression [23, 24]. Previous work has shown that the phosphorylation of pyruvate dehydrogenase complex, which catalyzes the entry of glycolytic products into the tricarboxylic acid cycle, was increased in calcified vessels of patients with atherosclerosis [25]. However, very rare studies have reported the precise function of OXPHOS in vascular calcification in CKD. For the first time, our data indicated that OXPHOS was significantly enhanced in high phosphate and uremic serum-induced calcification. We also found that the genes controlling the influx of pyruvate into mitochondria for OXPHOS were increased, which may help explain why OXPHOS was enhanced. Upregulating the level of OXPHOS exacerbated high phosphate-induced calcification. These results supported a key effect for OXPHOS in vascular calcification.

COX is the terminal complex of the mitochondrial respiratory chain, which catalyzes the transfer of electrons from cytochrome *c* to molecular oxygen [18]. Studies have indicated that COX serves as the rate-limiting step of the electron transport chain, which maintains tight control over OXPHOS flux and ATP production [26, 27]. Besides, previous studies have provided data supporting the positive relationship between COX and cancer progressions [27, 28]. In our study, transcriptome analysis revealed that COX was enriched in high phosphate-treated VSMCs, and significantly elevated levels of COX15 were found in artery tissues of both uremic patients and CKD mice. Moreover, the intensity of COX15 expression was positively associated with AAC. AAC is a strong indicator for both cardiovascular and non-cardiovascular mortality, which is independent of other cardiovascular risk factors and calcification elsewhere [29]. Numerous studies have consistently demonstrated that AAC is related to cardiovascular or all-cause mortality in CKD patients [30–32]. Therefore, it is important to identify whether the expression of COX15 is associated with the prognosis of CKD patients in future studies. In this study, we found that pharmacological inhibition of COX15 attenuated calcification. Current therapeutic options for vascular calcification in the clinical setting are scarce. Given an essential role of metabolic perturbation in vascular calcification, especially the increased expression of COX15 in artery tissues, we speculated that inhibition of COX15 might present a new strategy to delay the vascular calcification process.

This study has several limitations. First, our cohort is a retrospective study, a prospective study is required to further confirm the association of expression level of COX15 and cardiovascular or all-cause mortality in CKD patients. Second, we have shown that suppression of COX had a significant inhibitory effect on vascular calcification ex vivo, further in vivo studies might be necessary.

In summary, our study unravels a novel metabolic feature of calcified VSMCs, which revealed a preference for OXPHOS in glucose metabolism. The expression level of COX15 was positively related to AAC in CKD, and suppressing COX could significantly reduce vascular calcification. Our findings thus provide novel potential therapeutic strategies for treating vascular calcification in CKD.

## Materials and methods

### Reagents

Fasentin (HY-101849), etomoxir (HY-50202A), BPTES (HY-12683), ADDA 5 hydrochloride (HY-U00448), 2-DG (HY-13966), and cetylpyridinium chloride monohydrate (HY-B1289) were purchased from MedChemExpress (New Jersey, USA). The trizol reagent was purchased from Takara (Japan), and the reverse transcription system kit and SYBR Green PCR master mix kit were obtained from Vazyme (Nanjing, China). ATP assay kit (S0026), ROS assay kit (S0033S), and Mito-Tracker Green (C1048) were from Beyotime (Jiangsu, China). Calcium assay kit (C004-2-1), lactate assay kit (A019-2-1), and glucose assay kit (A154-1-1) were purchased from Nanjing Jiancheng (Nanjing, China). Cytochrome *c* oxidase activity kit (BC0940) were from Solarbio (Beijing, China). The antibody of COX15 (A14665) and β-actin (AC038) was obtained from Abclonal (Wuhan, China).

### Human samples

A cohort of 34 patients (61.8% men) with end-stage renal diseases undergoing arteriovenous fistula creation or arteriovenous graft placement was enrolled at Tongji Hospital from July 2020 to August 2021. The exclusion criteria were age <18 years old, diabetes, HIV, and malignant tumors. Informed written consent was obtained from all patients. The artery samples of the 34 patients were acquired during the arteriovenous fistula creation or arteriovenous graft placement. Radial artery samples of control patients (*n* = 5) underwent amputation surgery due to arm trauma with no medical history of diabetes or CKD were also acquired, CKD was defined as eGFR less than 60 mL/min per 1.73 m^2^, or markers of kidney damage (albuminuria, hematuria, or abnormalities detected through laboratory testing or imaging), or both, of at least 3 months duration [33]. Besides, the five patients in the control group did not have a medical history of stroke, ischemic heart disease, hypertensive heart disease, cardiomyopathy, atrial fibrillation, and aortic aneurysm. Moreover, blood samples were collected from hemodialysis patients before dialysis (uremic serum) and healthy individuals as controls (control serum). The AAC score is based on the degree of AAC on chest computed tomography (0 points, no visible calcification; 1 point, small spots or a single thin area of calcification; 2 points, 1 or more areas of thick calcification; 3 points, circumferential calcification) [29].

### Animal models

To induce CKD, male C57BL/6 mice underwent a two-step, 5/6 nephrectomy. In detail, mice were anesthetized in an induction chamber with 4% isoflurane and 100% oxygen, and maintained with 1.5% isoflurane. Mice underwent a total excision of the right kidney, one week later, both poles of the left kidney were resected, leaving an intact kidney segment which was returned to the abdominal cavity. The sham group received sham operations in which the appropriate kidney was exposed and mobilized but not treated in any other way. One week after the operation, mice were fed with 1.0% phosphorus diet for 4 weeks followed by 2.0% phosphorus diet for 8 weeks as previously described [34]. Mice were euthanized by overdose of isoflurane followed by cervical dislocation.

### Aortic ring treatment

Aortic tissues were removed from male C57BL/6 mice in a sterile manner. After the adventitia and adipose tissues were removed, the aortas were cut into 2–3-mm rings and cultured in Dulbecco’s Modified Eagle’s Medium (DMEM) containing 15% fetal bovine serum (FBS) at 37 °C in 5% CO_2_. To induce calcification, aortic rings were treated with high phosphate (3 mmol/L) for 14 days. The medium was replaced every 3 days.

### Cell culture and treatments

Mouse vascular smooth muscle cells (MOVAS) cell line and HASMCs were cultured in DMEM supplemented with 10% FBS. For calcification experiments, cells were treated with 3.0 mmol/L Pi for 24 h (RT-qPCR) or 3 days (calcium deposition quantification, and western blot), or 7 days (alizarin red staining). For ATP concentration, ROS, Mito-Tracker Green, and lactic acid concentration experiments, cells were treated with 3.0 mmol/L Pi for 24 h. Fresh media with agents were replaced every 2–3 days. For human serum study, HASMCs were treated with 2.5% uremic serum or control serum.

### Calcification analysis

The quantification of VSMCs calcification was performed by incubation of the VSMCs overnight at 4 °C in 0.6 M HCL. Calcium content was measured with calcium assay kit and the results were normalized to protein concentration. Alizarin red staining was performed in aortas and VSMCs. Paraformaldehyde-fixed thoracic aortic tissues were stained with 2% alizarin red solution after deparaffinization and rehydration. For VSMCs experiments, cells were fixed and then stained with 2% alizarin red solution. To quantify Alizarin red staining, we added hexadecylpyridinium chloride solution (100 mM) to each well and measured the optical density at 560 nm.

### Immunohistochemistry staining

Vascular tissues were fixed in paraformaldehyde and embedded in paraffin. After deparaffinization and rehydration, antigen retrieval was performed by citrate buffer (pH 6.0). Then, aortic sections were blocked with H_2_O_2_ for 15 min and blocked with goat serum for 1 h at room temperature, and then incubated overnight at 4 °C with primary antibodies against COX15 (1:100). Vascular sections were washed with PBST and then stained with horseradish peroxidase (HRP)-labeled secondary antibodies. Then, color was developed in all samples at the same time with 3,3-diaminobenzidine (DAB), and sections were counterstained with hematoxylin and visualized.

### Western blotting

Total protein from artery tissues and VSMCs were extracted in RIPA buffer supplemented with protease and phosphatase inhibitors. Proteins were separated by 10% sodium dodecyl sulfate-polyacrylamide gel electrophoresis (SDS-PAGE) and transferred to 0.45-μm polyvinylidene fluoride (PVDF) membranes. The membranes were blocked with 5% milk and then incubated with the following antibodies overnight at 4 °C: COX15 (1:1000) and β-actin (1:4000). Then, the membranes were washed by TBST and incubated with HRP-conjugated second antibodies (1:4000) for 1.5 h at room temperature, and the bound proteins were visualized by the enhanced chemiluminescence (ECL) method. The relative protein levels were calculated by normalizing to the loading control by using Image J.

### RT-qPCR

Total RNA was extracted from the aortas or VSMCs using Trizol reagent, and then reverse-transcribed into complementary DNA by the reverse transcription system kit. Each expression level was detected by the SYBR master mix. The mRNA expression level was normalized to GAPDH, which were measured as an internal control. For analysis of mtDNA levels, total DNA from HASMCs was amplified using primers specific for mitochondrially encoded cytochrome *c* oxidase 1 (mt-Co1) and normalized to β-2 macroglobulin (B2M) [35]. The designed primers are listed in Supplementary Table S1.

### ATP content and cytochrome *c* oxidase activity determination

The ATP contents were detected by ATP determination kit. In brief, samples (aortas or VSMCs) were lysed and centrifuged, then, the supernatants were collected and incubated with ATP detection working solution. The luminescence was measured and normalized to total protein content.

The activity of COX was measured according to the manufacturer’s instructions, by monitoring the absorbance of reduced cytochrome *c* at 550 nm and normalized to protein concentration.

### Measurement of glucose uptake and lactate production

A glucose assay kit was used to detect glucose uptake according to the manufacturer’s protocol. Lactate generation was determined using the lactate assay kit, for measurement of aortas, the results were normalized to the total protein concentration.

### Measurement of Mito-Tracker Green and ROS content

The measurement Mito-Tracker Green was determined using the assay kit according to the manufacturer’s protocol. In brief, cells were washed with PBS three times and incubated with 200 nM Mito-Tracker Green working solution for 40 min at 37 °C in the dark. After incubation, cells were washed with cell culture medium and the fluorescence intensity was measured. The intracellular ROS level was determined with the ROS assay kit. Cells were washed with PBS three times and incubated with 10 μM DCFH-DA for 20 min at 37 °C in the dark. After incubation, cells were washed with serum-free DMEM and the fluorescence intensity was measured.

### Transcriptome sequencing analysis

RNA samples were isolated from normal cultured or high phosphate-treated MOVAS. Sequencing was performed on the DNBSEQ platform. Then, the sequencing reads were processed with the determination of quality using the SOAPnuke tool. The clean reads were mapped to the reference genome using HISAT2 (v2.0.4). The heatmap was drawn by pheatmap (v1.0.8) according to the gene expression in different samples. Essentially, differential expression analysis was performed. To take insight to the change of phenotype, gene ontology (GO), Kyoto Encyclopedia of Genes and Genomes (KEGG) enrichment analysis, and gene set enrichment analysis (GSEA) of annotated differently expressed genes were performed. The significant levels of terms and pathways were corrected by *Q* value with a rigorous threshold (*Q* value ≤0.05) by Bonferroni.

### OCR analysis

HASMCs were treated with phosphate or human serum for 12 h for analysis of extracellular flux. An XF-24 Extracellular Flux Analyzer (Seahorse Bioscience, USA) was used for real-time analyses of OCR of HASMCs according to the manufacturer’s instruction. OCR was measured under basal conditions followed by the injection of oligomycin (1 μM), FCCP (1 μM), and rotenone (0.5 μM) plus antimycin (0.5 μM). The results were measured and normalized to total protein content.

### Statistical analysis

Statistical analysis was conducted using SPSS 22.0 software (SPSS, USA) and GraphPad Prism version 6 software (Graph software, USA). Continuous variables were displayed as mean ± standard error of mean or median with interquartile range. Categorical variables were expressed as frequencies and percentages. One-way ANOVA test, unpaired *t* test, or Mann–Whitney *U* test were used for continuous data as appropriate. Chi-square tests or Fisher’s exact tests were used for categorical variables. The number of samples for each data (*n*) is mentioned in figure legends. The statistical significance is expressed as follows: *P < 0.05; **P < 0.01; ***P < 0.001; ****P < 0.0001; and n.s., not significant.

## Supplementary information

Table S1

supplementary figure legend

Figure S1

Figure S2

Figure S3

checklist

change of authorship request form

## Data Availability

The data underlying this article will be shared on reasonable request to the corresponding author.
