# Supplementary material for: Oxidative phosphorylation promotes vascular calcification in chronic kidney disease
Source: Cell Death Dis. 2022 Mar 11;13(3):229. doi: 10.1038/s41419-022-04679-y (PMC8917188; doi:10.1038/s41419-022-04679-y)
Supplement: Supplementary file 1 — Table S1 [file 41419_2022_4679_MOESM1_ESM.docx]

Table S1. Primers used to amplify cDNA.

|  | Primers | Sequence (Sense/Antisense) |
| --- | --- | --- |
| mouse | Runx2 | 5′-AGCGGACGAGGCAAGAGTTT-3′ |
|  |  | 5′-AGGCGGGACACCTACTCTCATA-3′ |
|  | Sox9 | 5′-AGCTCTGGAGACTTCTGAACGAGA-3′ |
|  |  | 5′-ACTTGTAATCCGGGTGGTCCTTCT-3′ |
|  | Alpl | 5′-TTGTGCCAGAGAAAGAGAGAGA-3′ |
|  |  | 5′-GTTTCAGGGCATTTTTCAAGGT-3′ |
|  | Gapdh | 5′-CCTCGTCCCGTAGACAAAATG-3′ |
|  |  | 5′-TGAGGTCAATGAAGGGGTCGT-3′ |
| human | Nudfa1 | 5′-ATCCACAGGTTCACTAACGGGG-3′ |
|  |  | 5′-TAACGATCAACTCCAGAGATGCG-3′ |
|  | Nudfs1 | 5′-CTGGGAGCAGATGGAGGTTGTA-3′ |
|  |  | 5′-CTTGCCAAGCCAGGAGGTGT-3′ |
|  | Sdha | 5′-TGGGAACAAGAGGGCATCTG-3′ |
|  |  | 5′-CCACCACTGCATCAAATTCATG-3′ |
|  | Sdhb | 5′-TCAGGAAGGCAAGCAGCAGTATC-3′ |
|  |  | 5′-GCTCCTCTGTGAAGTCATCTCTGG-3′ |
|  | Uqcrc1 | 5′-GAGTACGGCAACCTTCGCTCA-3′ |
|  |  | 5′-ACGGCTGCCAACATCAATCC-3′ |
|  | Cox10 | 5′-TGCTGCTACACACCACTGAAA-3′ |
|  |  | 5′-TCAGGGCGTTGAAATGAGGA-3′ |
|  | Cox15 | 5′-TGAGTCCAGAACCCCTGTCA-3′ |
|  |  | 5′-GCTCGCCAGTGTTACTGTCT-3′ |
|  | ATP5g1 | 5′-CGCTGTTGTACCAGGGGTCTAA-3′ |
|  |  | 5′-GCTGTAGGAAGGCTGTTTAGATGA-3′ |
|  | Hk2 | 5′-GACTTCCGCACAGAATTTGATG-3′ |
|  |  | 5′-GAATGTTACGGACAATCTCACCC-3′ |
|  | Pdk1 | 5′-CTCAGGACACCATCCGTTCA-3′ |
|  |  | 5′-ATCTTGCAGGCCATACAGCA-3′ |
|  | Pkm2 | 5′-CGAGCCTCAAGTCACTCCACAG-3′ |
|  |  | 5′-AACATTCATGGCAAAGTTCACCC-3′ |
|  | Ldha | 5′-GAAGATAAGTGGTTTTCCCAAAAA-3′ |
|  |  | 5′-CTTTGAGTTTGATCACCTCATAAGC-3′ |
|  | Mpc1 | 5′-TCAGTGGGCGGATGACATTT-3′ |
|  |  | 5′-CTCGTGTTTGATAAGCCGCC-3′ |
|  | Mpc2 | 5′-GCAGGAGCCTCTCAGCTTTT-3′ |
|  |  | 5′-AGGTCCCAATGGTTTTGTCCA-3′ |
|  | Dlat | 5′-CAACAGCGTGACTACAGGGT-3′ |
|  |  | 5′-ATGGAACCTTCTGATGCGGG-3′ |
|  | Pdhb | 5′-TCCAGTGGTGGTGCTAGAGA-3′ |
|  |  | 5′-AGCACTGCTGCAGCTTCTAA-3′ |
|  | Runx2 | 5′-CACAAGTGCGGTGCAAACTT-3′ |
|  |  | 5′-GACTCTGTTGGTCTCGGTGG-3′ |
|  | Sox9 | 5′-AGGAAGTCGGTGAAGAACGG-3′ |
|  |  | 5′-AAGTCGATAGGGGGCTGTCT-3′ |
|  | Alpl | 5′-GGGACTGGTACTCAGACAACG-3′ |
|  |  | 5′-GTAGGCGATGTCCTTACAGCC-3′ |
|  | Gapdh | 5′-GGAAGCTTGTCATCAATGGAAATC-3′ |
|  |  | 5′-TGATGACCCTTTTGGCTCCC-3′ |
|  | Mt-Co1 | 5′-CCACCTCTAGCCTAGCCGTTTA-3′ |
|  |  | 5′-GGGTCATGATGGCAGGAGTAAT-3′ |
|  | B2M | 5′-TGCTGTCTCCATGTTTGATGTATCT-3′ |
|  |  | 5′-TCTCTGCTCCCCACCTCTAAGT-3′ |
