# Supplementary material for: Oxidative phosphorylation promotes vascular calcification in chronic kidney disease
Source: Cell Death Dis. 2022 Mar 11;13(3):229. doi: 10.1038/s41419-022-04679-y (PMC8917188; doi:10.1038/s41419-022-04679-y)
Supplement: Supplementary file 2 — supplementary figure legend [file 41419_2022_4679_MOESM2_ESM.docx]

**Supplementary figures**

**Fig. S1. High phosphate induced the calcification and osteoblast differentiation of VSMCs.** VSMCs were cultured with control or with high phosphate medium. (A) Representative images and quantification of Alizarin red staining of VSMCs (unpaired t-test; HASMC n=5, MOVAS n=4). (B) Total calcium content in VSMCs (unpaired t-test; HASMC n=4, MOVAS n=6). (C) Relative mRNA expression of Runx2, Sox9, ALPL in VSMCs as detected by RT-qPCR (unpaired t-test; HASMC n=6, MOVAS n=4). *P < 0.05, **P < 0.01, ****P < 0.0001.

**Fig. S2. 5/6 nephrectomy plus high phosphate diet induced calcification in aortas of CKD mice.** (A) Serum creatinine, blood urea nitrogen, and serum phosphate concentrations in mice treated with sham-operated or 5/6 nephrectomy plus high phosphate diet (unpaired t-test; sham n=5, 5/6 nephrectomy n=6). (B) Representative images of Alizarin red staining of aortic arch sections from mice. (C) Total calcium content in aortas in mice (unpaired t-test; sham n=5, 5/6 nephrectomy n=6). (D) Relative mRNA expression of Runx2, Sox9, ALPL in aortic tissues as detected by RT-qPCR (unpaired t-test; sham n=5, 5/6 nephrectomy n=6). *P < 0.05, **P < 0.01, ****P < 0.0001. Scale bar = 20 µm.

**Fig. S3. Uncropped scans of western blots for Fig.5G and 5I, Fig.7B.**
