# Supplementary figures and images for: Oxidative phosphorylation promotes vascular calcification in chronic kidney disease
Source: Cell Death Dis. 2022 Mar 11;13(3):229. doi: 10.1038/s41419-022-04679-y (PMC8917188; doi:10.1038/s41419-022-04679-y)

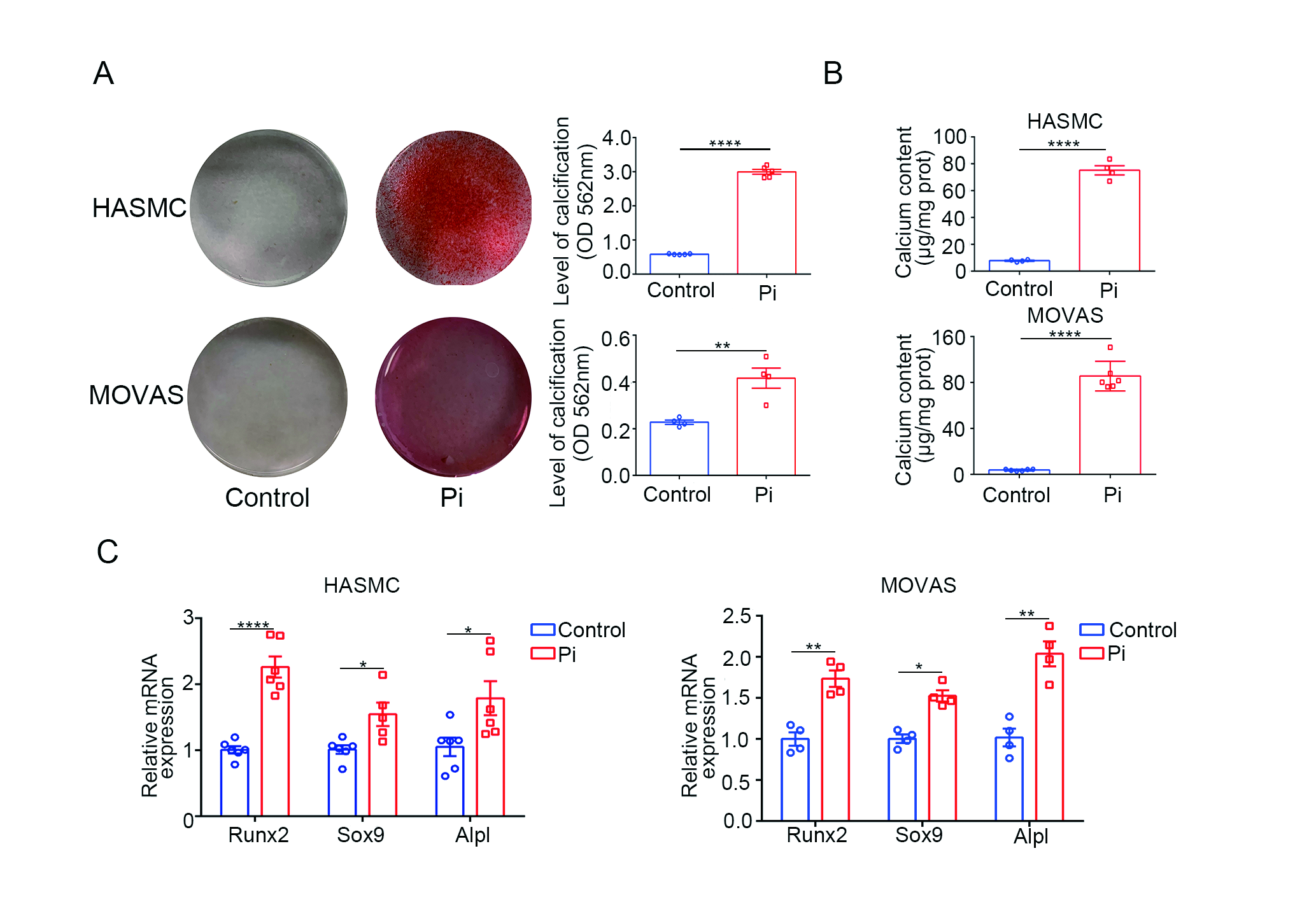

Supplement: Supplementary file 3 — Figure S1 [file 41419_2022_4679_MOESM3_ESM.tif]

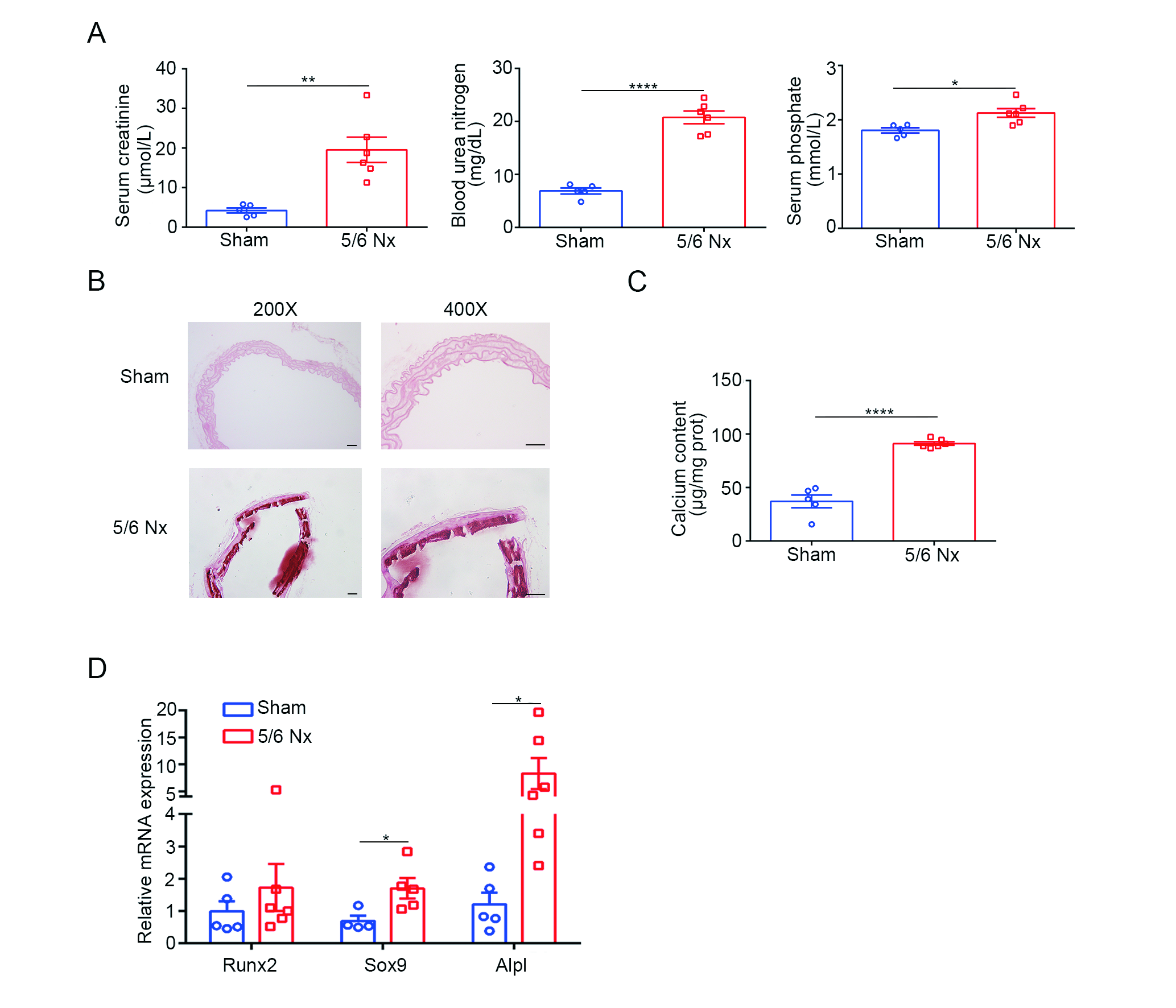

Supplement: Supplementary file 4 — Figure S2 [file 41419_2022_4679_MOESM4_ESM.tif]

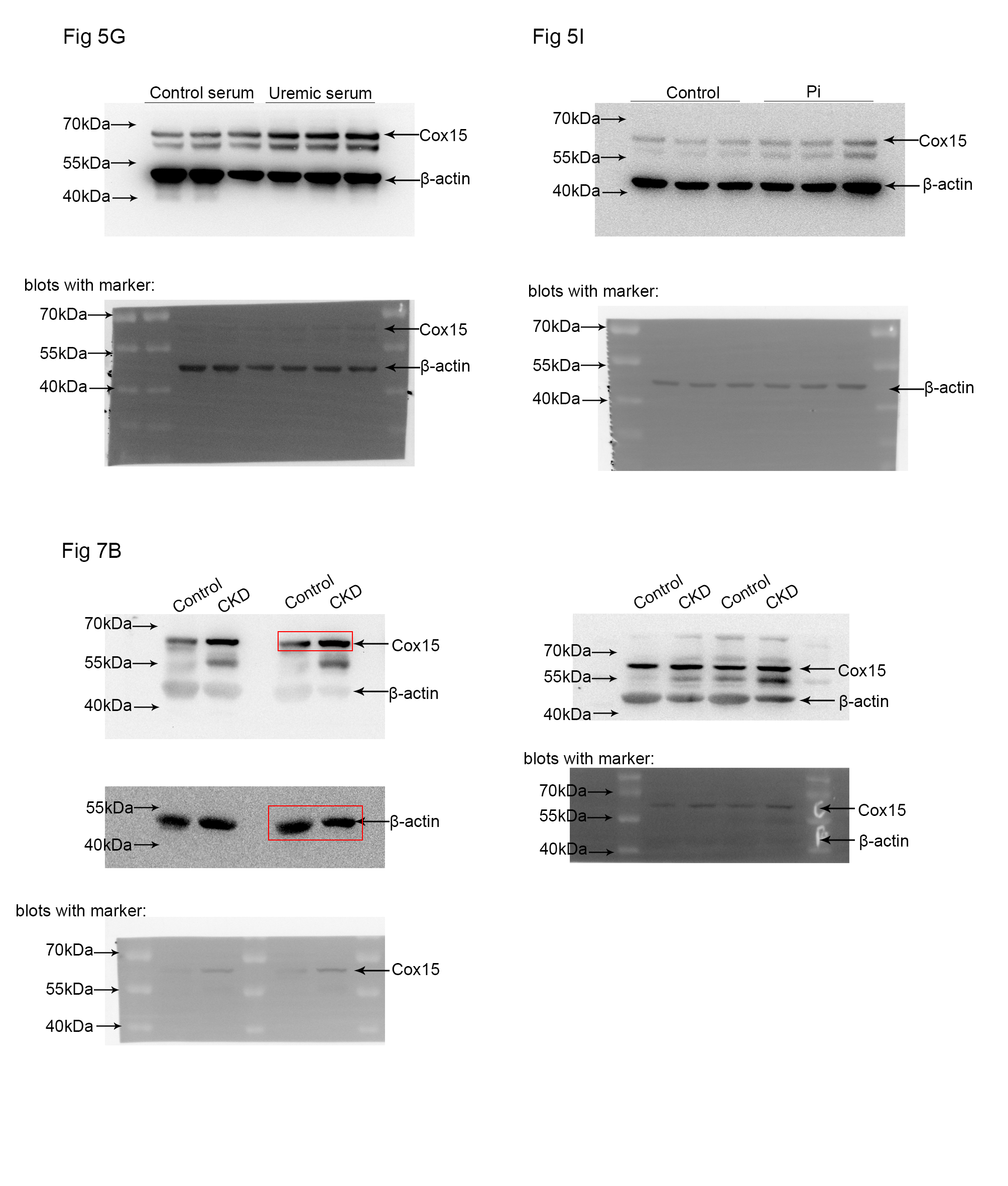

Supplement: Supplementary file 5 — Figure S3 [file 41419_2022_4679_MOESM5_ESM.png]
