# Supplementary material for: Oxidative phosphorylation promotes vascular calcification in chronic kidney disease
Source: Cell Death Dis. 2022 Mar 11;13(3):229. doi: 10.1038/s41419-022-04679-y (PMC8917188; doi:10.1038/s41419-022-04679-y)
Supplement: Supplementary file 7 — change of authorship request form [file 41419_2022_4679_MOESM7_ESM.pdf]

SPRINGER NATURE

Change of authorship request form - Journals (pre-acceptance)

Important information. Please read.

- This form should be used by authors to request any change in authorship (adding/deleting authors) including changes in corresponding authors. This form should not be used for name changes. Please fully complete all sections. Use black ink and block capitals and provide each author's full name with the given name first followed by the family name.
- By signing this declaration, all authors guarantee that the order of the authors are in accordance with their scientific contribution, if applicable as different conventions apply per discipline, and that only authors have been added who made a meaningful contribution to the work.
- Please note, in author collaborations where there is formal agreement for representing the collaboration, it is sufficient for the representative or legal guarantor (usually the corresponding author) to complete and sign the Authorship Change Form on behalf of all authors, **next to the added/removed author(s). (Complete Section 3, followed by Section 6.)**
  In author collaborations where there is no formal agreement for representing the collaboration and **there are more than 10 authors**, one may sign for all, provided the signer appends correspondence that attests that each of the authors have agreed to the change **and the added/removed authors sign the form. (Complete Section 3, followed by Section 6.)**
- Please note, we cannot investigate or mediate any authorship disputes. If you are unable to obtain agreement from all authors (including those who you wish to be removed) you must refer the matter to your institution(s) for investigation. Please inform us if you need to do this.
- If you are not able to return a fully completed form within **30 days** of the date that it was sent to the author requesting the change, we may have to withdraw your manuscript. We cannot publish manuscripts where authorship has not been agreed by all authors (including those who have been removed).
- Incomplete forms will be rejected.
- Please return/upload this form, fully completed, to the Journals Editorial Office. The Journal and/or Publisher will consider the information you have provided to decide whether to approve the proposed change in authorship. We may decide to contact your institution for more information or undertake a further investigation, if appropriate, before making a final decision.

SPRINGER NATURE

Change of authorship request form - Journals (pre-acceptance)

Section 1: Please provide the current title of manuscript

Manuscript ID no.: CDDIS-21-4562

Title: Oxidative phosphorylation promotes vascular calcification in chronic kidney disease

Section 2: Please provide the previous authorship, in the order shown on the manuscript before the changes were introduced. Please indicate the corresponding author by adding (CA) behind the name.

	First name(s)	Family name	ORCID or SCOPUS id, if available
1st author	JIA	SHI	
2nd author	YI	YANG	
3rd author	QING	LI	
4th author	XUE	XING	
5th author	AN-YING	CHENG	
6th author	YA-NAN	WANG	
7th author	XIAO-NA	ZHAN	
8th author	JIE	LI	
9th author	GANG (CA)	XU (CA)	
10th author	FAN (CA)	HE (CA)	

Please use an additional sheet if there are more than 10 authors.

SPRINGER NATURE Springer Nature is one of the world's leading global research, educational and professional publishers, created in May 2015 through the combination of Nature Publishing Group, Palgrave Macmillan, Macmillan Education and Springer Science+Business Media.

SPRINGER NATURE

Change of authorship request form - Journals (pre-acceptance)

Section 3: Please provide a justification for change. Please use this section to explain your reasons for changing the authorship of your manuscript, e.g. what necessitated the change in authorship? Please refer to the (journal) policy pages for more information about authorship. Please explain why omitted authors were not originally included and/or why authors were removed on the submitted manuscript.

Ya-Nan Wang did a lot of works in the revisement of manuscript, so we changed the order of authorship.

Section 4: Proposed new authorship. Please provide your new authorship list in the order you would like it to appear on the manuscript. Please indicate the corresponding author by adding (CA) behind the name. If the Corresponding Author has changed, please indicate the reason under section 3.

	First name(s)	Family name (this name will appear in full on the final publication and will be searchable in various abstract and indexing databases)	Affiliated institute	E-mail address
1st author	JIA	SHI	Tongji Hospital, Tongji Medical College, Huazhong University of Science and Technology	shijia94@foxmail.com
2nd author	YI	YANG	Tongji Hospital, Tongji Medical College, Huazhong University of Science and Technology	yiyang@tjh.tjmu.edu.cn
3rd author	YA-NAN	WANG	Tongji Hospital, Tongji Medical College, Huazhong University of Science and Technology	wynan1996@163.com
4th author	QING	LI	Tongji Hospital, Tongji Medical College, Huazhong University of Science and Technology	liqing_med@outlook.com
5th author	XUE	XING	Tongji Hospital, Tongji Medical College, Huazhong University of Science and Technology	xxbss123@126.com
6th author	AN-YING	CHENG	Tongji Hospital, Tongji Medical College, Huazhong University of Science and Technology	biocay@163.com
7th author	XIAO-NA	ZHAN	Tongji Hospital, Tongji Medical College, Huazhong University of Science and Technology	zhanxiaona1997@163.com
8th author	JIE	LI	Tongji Hospital, Tongji Medical College, Huazhong University of Science and Technology	lijiee1997@foxmail.com
9th author	GANG (CA)	XU (CA)	Tongji Hospital, Tongji Medical College, Huazhong University of Science and Technology	xugang@tjh.tjmu.edu.cn
10th author	FAN (CA)	HE (CA)	Tongji Hospital, Tongji Medical College, Huazhong University of Science and Technology	fhe@tjh.tjmu.edu.cn

Please use an additional sheet if there are more than 10 authors.

SPRINGER NATURE Springer Nature is one of the world's leading global research, educational and professional publishers, created in May 2015 through the combination of Nature Publishing Group, Palgrave Macmillan, Macmillan Education and Springer Science+Business Media.

SPRINGER NATURE **Change of authorship request form - Journals** (pre-acceptance)

Section 5: Author contribution, Acknowledgement and Disclosures. Please use this section to provide a new disclosure statement and, if appropriate, acknowledge any contributors who have been removed as authors and ensure you state what contribution any new authors made (if applicable per the journal or book (series) policy). Please ensure these are updated in your manuscript - after approval of the change(s) - as our production department will not transfer the information in this form to your manuscript.

New acknowledgements:

Not applicable

New Disclosures (financial and non-financial interests, funding):

Not applicable

New Author Contributions statement (if applicable per the journal policy):

FH, GX, and JS designed the study; JS, YY, YNW, QL, XX, and AYC performed the experiments and analyzed the data; JS, YNW and JL provided patient materials; JS and XNZ drafted the manuscript; FH and GX revised the manuscript; all authors approved the final version of manuscript.

State 'Not applicable' if there are no new authors.

SPRINGER NATURE

Change of authorship request form - Journals (pre-acceptance)

Section 6: Declaration of agreement. *All* authors, unchanged, new and removed *must* sign this declaration.
(NB: Please print the form, (docu)-sign and return/upload a scanned copy. Please note that signatures that have been inserted as an image file are acceptable as long as it is handwritten. Typed names in the signature box are unacceptable.) *** Please delete as appropriate. Delete all of the bold if you were on the original authorship list and are remaining as an author.**

	First name	Family name		Signature	Date
1st author	JIA	SHI	I agree to the proposed new authorship shown in section 4 **/and the addition/removal*of my name to the authorship list** /and the proposed change in corresponding author	Jia Shi	2022.2.10
2nd author	YI	YANG	I agree to the proposed new authorship shown in section 4 **/and the addition/removal*of my name to the authorship list** /and the proposed change in corresponding author	Yi Yang	2/10/2022
3rd author	YA-NAN	WANG	I agree to the proposed new authorship shown in section 4 **/and the addition/removal*of my name to the authorship list** /and the proposed change in corresponding author	Ya Nan Wang	2022.2.10
4th authors	QING	LI	I agree to the proposed new authorship shown in section 4 **/and the addition/removal*of my name to the authorship list** /and the proposed change in corresponding author	Qing Li	2022.2.10
5th author	XUE	XING	I agree to the proposed new authorship shown in section 4 **/and the addition/removal*of my name to the authorship list** /and the proposed change in corresponding author	Xue XING	2022.2.10
6th author	AN-YING	CHENG	I agree to the proposed new authorship shown in section 4 **/and the addition/removal*of my name to the authorship list** /and the proposed change in corresponding author	AnYing Cheng	2/10/2022
7th author	XIAO-NA	ZHAN	I agree to the proposed new authorship shown in section 4 **/and the addition/removal*of my name to the authorship list** /and the proposed change in corresponding author	Xiaona Zhan	2022.2.10

SPRINGER NATURE **Change of authorship request form - Journals (pre-acceptance)**

	First name	Family name		Signature	Date
8th author	JIE	LI	I agree to the proposed new authorship shown in section 4 **/and the addition/removal*of my name to the authorship list** /and the proposed change in corresponding author	JIE LI	2/10/2022
9th author	GANG	XU	I agree to the proposed new authorship shown in section 4 **/and the addition/removal*of my name to the authorship list** /and the proposed change in corresponding author	Gang Xu	2/10/2022
10th author	FAN	HE	I agree to the proposed new authorship shown in section 4 **/and the addition/removal*of my name to the authorship list** /and the proposed change in corresponding author	Fan He	2/10/2022

Please use an additional sheet if there are more than 10 authors.

In case of author collaborations with formal agreement:

	Name of consortium/consortia	First name	Family name		Signature	Date
Representative/legal guarantor				I agree to the proposed new authorship shown in section 4 **/and the addition/removal*of my name to the authorship list** /and the proposed change in corresponding author		

Both added/removed authors should complete the information in the first table under Section 6.

---- End of form ----

SPRINGER NATURE Springer Nature is one of the world's leading global research, educational and professional publishers, created in May 2015 through the combination of Nature Publishing Group, Palgrave Macmillan, Macmillan Education and Springer Science+Business Media.
